# Annotations

**Published:** 1910-08-13

**Authors:** 


### ANNOTATIONS.

### The Paris Pharmacists' Shorter Hours Movement.

The necessity of being able to obtain medicaments at all hours is recognised by the medical fraternity and the nursing sisterhood. But the majority thereof, being themselves worked so hardly, can scarcely fail to sympathise with the retail chemist on account of his lengthy hours of business attendance. Perhaps, however, a labour-saving scheme for the pharmacist which is now being organised in Paris may be copied in parts of the United Kingdom before long. The Paris chemists are arranging to close their establishments at 9 P.M. and not to reopen until 8 A.M. But to meet public requirements between those hours a co-operative pharmacy will be started and maintained by them in each *arrondissement*. This will be open during the closing-time indicated. All the chemists of each *arrondissement* will have an interest in it, and will supply the necessary service in turn. Notices will appear inside and outside their own shops indicating the position of each "open all night and early morning" co-operative pharmacy. It has been arranged that a committee shall supervise the conformity to the closing agreement, and any infringement of it will be visited by a penalty of 100 francs. Three pharmacists only in the twenty *arrondissements* have (so states the *Figaro*) declined to close their establishments at 9 P.M. Arrangements will also be made to keep open the co-operative pharmacy all the Sunday during the summer months, so as to allow the other pharmacies to be closed meanwhile.

### Flannelette.

The report of the British Fire-Prevention Committee with reference to Fire Tests with Textiles * is to hand, and contains an elaborate account of the tests applied in determining the relative inflammability of the various fabrics examined. These last were divided into six groups: A, Commercial "non-flam" flannelette; B, special "non-flam" flannelette; C, ordinary cotton flannelette; D, union, a mixture of cotton and wool; E, all wool flannel; F, cotton flannelette, known as "fine surface." Each group was subdivided and dealt with, so that one set of samples of each was tested: (1) as received from the manufacturer; (2) after being once washed; (3) after five washings; (4) after ten washings; and (5) after twenty washings. A number of "demonstration tests" were also conducted with garments made up of materials described under groups A and C, and washed in each case five and ten times. The thirty-six samples of group A all passed the required standard to receive the classification of "non-flaming." Only a smouldering occurred after the flame was removed, and less than 2 per cent. of any of the samples was burnt when a taper was applied to them, and less than 4 per cent. after the application of a spirit-lamp. The fire-resistance was little changed by the number of washings. Even better results were obtained with Group B samples. The results obtained with Group C samples showed the extraordinary rapidity with which untreated flannelette burns. In sixty seconds an average of 33 per cent. of the material was consumed in unwashed samples, an average which jumped to 97 per cent. in washed ones. In some cases the whole of the material was consumed by the flame. The results obtained from testing Group D were somewhat better than this, an average of 35 per cent. of the material being consumed in unwashed and 50 per cent. in washed samples in sixty seconds, none of the samples being consumed entirely. Group E, flannel, obtained the classification of "non-flaming," there being no practical difference between washed and unwashed samples. The "fine finish" flannelette of Group F gave results very similar to those given by ordinary flannelette. From the results of the tests it will thus be seen that both brands of "non-flam" flannelette and all wool flannel are materials which it is safe to use. The Committee, as the result of their investigations, advise that for a material intended to be permanently non-inflammable the conditions of the following test must be carried out for it to obtain classification as "non-flaming": (*a*) Three samples each comprising one yard of material, shall be washed with soap and water and ironed ten times; (*b*) the samples shall be ironed once, in addition, with an ordinary household iron, not more than three hours and not less than one hour before the test, and shall be dry to the touch immediately before testing; (*c*) the samples thus prepared shall be measured exactly, and their areas shall not vary more than 10 per cent. above or below a square yard; (*d*) the samples shall be suspended in rotation from a wooden lath, vertically, by three tacks, clips, or other metal fastenings; (*e*) fire shall be applied at the centre of the bottom edge from a taper ⅛th inch in diameter, not more than 12 inches or less than 6 inches long; (*f*) the lighted end of the taper shall be held at the edge for not less than fifteen or more than thirty seconds; (*g*) if not more than 5 per cent. of the area actually under test burns within sixty seconds, when taken on the average of three samples, the material shall be classified as "non-flaming." In addition, a number of suggestions are made as to the legislation which should be introduced to deal with the marking of cotton, flannelette, and unions in such a way that each type can be readily recognised: with the selling of such goods under a false description, with the prohibition of the importation of foreign goods that do not comply with the "non-flaming" tests, with parents' and guardians' responsibility in case of death or injury from wearing inflammable flannelette and unions, and with the duties of public authorities in making known the dangers of untreated flannelette used as clothing material.

* Fire Tests with Textiles—Flannelette known as "non-flam," ordinary flannelette, "union," and flannel submitted for test by Messrs. Whipp Brothers and Tod, Ltd., Manchester. The British Fire Prevention Committee's Report. London: 1910. Price 5s.

